# *Broussonetia papyrifera* Polysaccharide Alleviated Acetaminophen-Induced Liver Injury by Regulating the Intestinal Flora

**DOI:** 10.3390/nu14132636

**Published:** 2022-06-25

**Authors:** Baichang Xu, Kaiyuan Hao, Xiaogang Chen, Enyun Wu, Dongyang Nie, Geyin Zhang, Hongbin Si

**Affiliations:** State Key Laboratory for Conservation and Utilization of Subtropical Agro-Bioresources, College of Animal Science and Technology, Guangxi University, Nanning 530004, China; 1918393057@st.gxu.edu.cn (B.X.); 1918302008@st.gxu.edu.cn (K.H.); 1918393006@st.gxu.edu.cn (X.C.); 1918302031@st.gxu.edu.cn (E.W.); 1918302023@st.gxu.edu.cn (D.N.); 2118402012@st.gxu.edu.cn (G.Z.)

**Keywords:** *Broussonetia papyrifera* polysaccharide, liver injury, intestinal flora

## Abstract

Liver injury caused by an overdose of acetaminophen (APAP) is a major public health problem. This study aimed to evaluate the effects of *Broussonetia papyrifera* polysaccharide (BPP) on liver injury and intestinal flora induced by APAP. The results showed that BPP could protect against APAP-induced liver injury, alleviate liver apoptosis, improve antioxidant capacity and enhance the liver’s detoxification ability to APAP. At the same time, BPP improved the intestinal flora disorder caused by APAP. More importantly, we found that the hepatoprotective effect of BPP disappeared after the depletion of gut microbiota in mice. Further, we reconstructed the intestinal flora structure of mice through fecal microbiota transplantation and found that the symptoms of APAP—induced liver injury were effectively alleviated. Overall, BPP was a potential hepatoprotective drug that could protect against APAP-induced liver injury and might be mediated by intestinal flora.

## 1. Introduction

Acetaminophen (APAP) is a widely used analgesic and antipyretic drug, but excessive APAP could lead to serious hepatotoxicity. It reported that APAP is the primary drug causing drug-induced liver injury (DILI) in western countries, and nearly 500 people die yearly in the United States [1]. In addition, non-alcoholic fatty liver disease, obesity, alcoholism, and chronic liver disease will increase the risk of acetaminophen poisoning [2]. It could be predicted that there will be more events of DILI caused by acetaminophen. N-acetyl-L-cysteine (NAC) is the only drug authorized for treatment of APAP poisoning in the clinic. However, the use of NAC is complicated due to its limited administration window, and its many serious side effects [3]. Therefore, finding a more effective treatment with low side effects is vital.

APAP is mainly converted into non-toxic metabolites by UDP-glucuronosyltransferases (UGTs) and sulfotransferases (SULTs) and then discharged from the body. Less than 10% of APAP is metabolized to toxic *N*-acetyl-p-benzoquinone imine (NAPQI) through cytochrome P450 (mainly CYP2E1) and further detoxified by combining with glutathione (GSH) under the action of glutathione transferase (GST) [4]. Excessive intake of APAP will exhaust GSH, resulting in a considerable accumulation of NAPQI in the liver. NAPQI can react with sulfhydryl groups to form protein adducts, damage mitochondrial function, cause cell death, and lead to abnormal liver function. Furthermore, the absence of GSH will cause intracellular oxidative stress and free radical production, aggravate cell death and cell content release, recruit more inflammatory cells to the damaged area of the liver, and finally lead to inflammatory reaction and secondary liver injury.

Historically, the hepatotoxicity of APAP has been considered the leading cause of liver damage. However, recent evidence suggested that changes in intestinal microbiota are closely related to APAP poisoning. Individuals with intestinal flora imbalance are more sensitive to liver injury caused by APAP [5]. More importantly, the symptoms of APAP liver injury in mice recurred when their fecal bacteria were transplanted into healthy mice [5,6]. Similarly, the changes in intestinal flora diversity and abundance could also affect the metabolism of APAP. Vancomycin decreased the bioavailability of APAP after changing the composition of intestinal microflora in mice, thereby alleviating the liver injury of APAP [7]. The pharmacokinetics of APAP was also affected by the changes in intestinal bacterial composition in mice. Studies have shown that adding probiotics could reduce the absorption of acetaminophen by changing the metabolism of microorganisms [8]. In addition, intestinal microorganisms play an important role in the expression and metabolism of P450 enzymes involved in liver APAP metabolism [9]. Moreover, intestinal microbiota and their metabolites can regulate oxidative stress and inflammation, which is closely related to DILI [10]. In conclusion, intestinal flora might play an essential role in liver injury induced by APAP.

In recent years, the development of the medicinal value of natural polysaccharides has become a research hotspot. A variety of plant polysaccharides have been proved to improve the intestinal flora environment, reduce intestinal bacterial translocation, inhibit liver inflammation, and reduce liver injury [11,12,13]. *Broussonetia papyrifera* is widely distributed in East and South Asia. Its leaves, fruits, and root bark are considered traditional drugs [14]. In addition, its leaves are rich in protein, amino acids, and other nutrients, which can be used to feed animals. It was found that various extracts and pure compounds of *Broussonetia papyrifera* have the characteristics of anti-oxidation, anti-inflammatory, and anti-hepatotoxicity [15,16,17]. We discovered that *Broussonetia papyrifera* polysaccharides (BPP) extracted from leaves could protect mice from liver damage caused by APAP. 16S rDNA sequencing showed that BPP could alleviate the liver damage and intestinal flora abnormalities caused by APAP. In addition, we also demonstrated that the protection of the liver by BPP depends on the intestinal flora, by depleting the intestinal flora and by fecal microbiota transplantation (FMT).

## 2. Materials and Methods

### 2.1. Materials and Reagents

Fresh *Broussonetia papyrifera* leaves were collected at the planting base (Beihai, China). Monosaccharide standard was purchased from BoRui Saccharide Biotech Co., Ltd. (Yangzhou, Jiangsu, China). Acetaminophen (AR, 99.0%) was purchased from Shanghai Aladdin Biochemical Technology Co., Ltd. (Shanghai, China). Bifendate Pills were purchased from Beijing Xiehe pharmaceutical factory (Beijing, China). Neomycin sulfate, Ampicillin sodium salt, Metronidazole, and Vancomycin hydrochloride were purchased from Macklin Biochemical Co., Ltd. (Shanghai, China). AST and ALT test kits were purchased from URIT Medical Electronics Co., Ltd. (Guilin, China). H&E and Hoechst33258 dye solution set were obtained from Servicebio technology Co., Ltd. (Wuhan, China). SOD, MDA, GSH, and GSH-PX test kits were purchased from Jiancheng Bioengineering Institute (Nanjing, China). Mice IL-6 (MM-0163M1), IL-10 (MM-0176M1), TNF-α (MM-0132M1), CYP2E1 (MM-44419M1), SULT1A1 (MM-0874M1), UGT (MM-0770M1), caspase-3 (MM-43676M1), caspase-8 (MM-45746M1), and caspase-9 (MM-1179M1) ELISA kits were purchased from Jiangsu Meimian industrial Co., Ltd. (Yancheng, Jiangsu, China). Spin Column Animal Total RNA Purification Kit was purchased from Sangon BiotechCo., Ltd. (Shanghai, China), First-strand cDNA Synthesis Mix and RealStar Green Fast Mixture were purchased from GenStar Co., Ltd. (Beijing, China).

### 2.2. Extraction, Separation and Purification of BPP

The fresh *Broussonetia papyrifera* leaves were washed, dried naturally, and crushed to 40 mesh. The powder was soaked in petroleum ether and ethanol for 12 h 3 times. Dry powder was added with ten times the water weight and extracted three times at 100 °C for 1 h each time. We collected all extracts, concentrated them to an appropriate volume, added four times the volume of absolute ethanol, and stored them at 4 °C for 12 h. The crude polysaccharides were collected by centrifugation at 4000 rpm for 10 min. The protein in polysaccharides was removed according to the reported method [18]. Then, the fat in the polysaccharide was removed with acetone, absolute ethanol, and absolute ether, and pure BPP was obtained by freeze-drying.

### 2.3. Structural Characterization of BPP

#### 2.3.1. Polysaccharide Content and Purity of BPP

Total carbohydrate content in BPP was determined by Sulfuric Acid-Phenol Method [19]. Then the BPP was scanned in the ultraviolet spectrophotometer (Thermo Fisher, Belmont, MA, USA) at the wavelength of 200–400 nm to judge whether the BPP contained nucleic acid and protein [20,21].

#### 2.3.2. FT-IR Analysis

2 mg BPP and 200 mg KBr were compressed and placed in a Fourier transform infrared spectrometer FI-IR650 (Tianjin Gangdong Sci. & Tech. Co., Ltd., Tianjin, China) for scanning and recording 4000 to 400 cm^−1^ [22].

#### 2.3.3. Monosaccharide Composition

Referring to the reported method, the monosaccharide components in BPP were determined by ion chromatogram ICS5000 (Thermo Fisher, Belmont, MA, USA) [23]. Standards of 17 monosaccharides were selected as reference, including Fucose (Fuc), Galactosamine hydrochloride (GalN), Rhamnose (Rha), Arabinose (Ara), Glucosamine hydrochloride (GlcN), Galactose (Gal), Glucose (Glc), *N*-acetyl-d-glucosamine (GlcNAc), *N*-acetyl-d-galactosamine (GalNAc), Xylose (Xyl), Mannose (Man), Fructose (Fru), Ribose (Rib), Galacturonic acid (GalA), Glucuronic acid (GulA), Glucuronic acid (GlcA), and Mannuronic acid(ManA).

### 2.4. Gut Microbiota Depletion and Fecal Microbiota Transplantation (FMT)

#### 2.4.1. Gut Microbiota Depletion

According to the reported method, mixed antibiotics were used to remove intestinal flora [24,25]. Briefly, vancomycin (100 mg/kg), neomycin sulfate (200 mg/kg), metronidazole (200 mg/kg), and ampicillin (200 mg/kg) were mixed in sterile water and gavaged once a day for 7 consecutive days to remove intestinal flora.

#### 2.4.2. Fecal Microbiota Transplantation (FMT)

Refer to the previous method, with appropriate adjustments: we collected the feces from donor mice (H-BPP) and resuspended them in sterile saline at 0.1 g/mL. Then, the fresh fecal suspension was gavaged according to 0.2 mL per mouse once a day for seven days [24].

### 2.5. Animals and Experimental Design

Male KM mice (4–6 weeks,18–22 g) were purchased from Changsha Tianqin Biotechnology Co., Ltd. (Changsha, China, License number: SCXK[Xiang]2019-0014). All mice lived at (22 ± 2) °C for 12 h of light-dark cycle and drank water and standard food freely. All animal experimental protocols were approved by the Institutional Animal Care and Use Committee of Guangxi University (Nanning, China) [No. Gxu-2021-173].

I. Mice were randomly divided into 6 groups (*n* = 10 for each group): control group (CON), APAP model group (APAP), low dose group (L-BPP), middle dose group (M-BPP), high dose group (H-BPP) and positive drug group (PD). For the next 14 days, mice in the CON and APAP groups were administered sterile water daily, while the L-BPP, M-BPP and, H-BPP groups were administered 100, 200, and 400 mg/kg/d BPP. Mice in the PD group were given 200 mg/kg/d Bifendate pills. After the last administration, all mice did not eat for 16 h. Then, mice in all groups except the CON group were gavaged with 200 mg/kg APAP, and the CON group was gavaged with an equal volume of sterile water. This APAP-induced liver injury model is considered stable and reliable [26,27]. We ensured that the mice did not eat within 12 h after taking APAP and then collected samples.

II. To further confirm that BPP improved APAP-induced liver injury by affecting intestinal microbiota, an FMT experiment was conducted. Mice were randomly divided into 4 groups (*n* = 8 for each group): control group (CON), APAP model group (APAP), antibiotic mixture treatment group (ABX), and FMT-treated group (FMT). ABX and FMT groups were treated with antibiotics according to the method described in Section 2.4.1, and the other mice were gavaged with sterile water. Then, the FMT group was perfused with fecal supernatant for 7 days, and the other mice were gavaged with sterile saline according to the method described in Section 2.4.2. On the last day of fecal microbiota transplantation, the feces of mice in each group were collected for 16S rDNA sequencing. The mice in APAP, ABX and FMT groups were induced with liver injury according to the method described in Section 2.5.I. We ensured that the mice did not eat within 12 h after taking APAP and then collected samples.

III. We designed the following experiments to confirm that BPP depended on intestinal microbiota to protect mice from liver injury caused by APAP. Mice were randomly divided into 3 groups (*n* = 8 for each group): control group (CON), antibiotic mixture treated model group (ABX), and antibiotic mixture and BPP treated group (ABX-BPP). ABX and ABX-BPP groups were treated with antibiotics according to the method described in Section 2.4.1, and the other mice were gavaged with sterile water. Then, mice in the ABX-BPP group were gavaged with 400 mg/kg BPP for 14 days, and other groups were gavaged with the same dose of sterile water. On the last day of gavage, mice in ABX and ABX-BPP groups were induced with liver injury according to the method described in Section 2.5 I. We ensured that the mice did not eat within 12 h after taking APAP and then collected samples.

The livers were isolated and weighed, and calculated using the following formula:

Liver index (%) = Liver weight (g)/Body weight (g) × 100%

### 2.6. Serum Biochemical Detection

After weighing, the mice were anesthetized and we took blood from the orbital sinus. The blood was stored at 4 °C for 12 h, then centrifuged at 3000 rpm for 15 min to obtain serum. The contents of AST and ALT were analyzed by the automatic biochemical analyzer URIT-8021Vet (URIT Medical Electronics Co., Ltd., Guilin, China).

### 2.7. Histological Analysis

The left lobe of the mice’s liver was fixed with 10% formalin. Then, we made paraffin sections according to the conventional histological method and performed H&E staining.

### 2.8. Determination of Liver Oxidative Stress Indicators

0.9% normal saline refrigerated at 4 °C was mixed with liver tissue according to the ratio of 9:1 to prepare liver homogenate, and the supernatant was obtained by centrifugation. According to the instructions, we determined the contents of SOD, GSH, MDA, and GSH-Px.

### 2.9. Determination of Cytokines, Metabolic Enzymes and Apoptosis-Related Proteins in Liver Tissue

We used the corresponding commercialized ELISA kit to determine the content or activity of inflammatory factors (IL-6, IL-10, and TNF-α), metabolic enzymes (CYP2E1, SULT1A1, and UGT), and apoptosis-related proteins (Caspase-3, Caspase-8, and caspase-9) in the liver.

### 2.10. Hoechst 33258 Staining

According to the production process of histopathological sections, after dewaxing and rehydration, PBS was used for repeated cleaning, and Hoechst 33258 staining was performed [28].

### 2.11. RNA Extraction and qRT-PCR Analysis

We weighed and ground 40–50 mg of mouse liver tissue, and extracted RNA with the Spin Column Animal Total RNA Purification Kit; then, RNA was detected by ultramicro ultraviolet spectrophotometer and 0.1% agarose gel electrophoresis. To ensure the quality and concentration of cDNA, we used the starscript II first-strand cDNA synthesis premix system (including gDNA remover) to remove genomic DNA. The purified RNA was used as a template to synthesize cDNA according to the instructions. According to the instructions of the RealStar Green Fast Mixture kit, the mRNA expression of liver-related proteins in mice was detected with a 20 µL system on the LightCycler 480 II System (Roche, Basel, Switzerland). We chose the β-Actin as an internal reference gene and calculated by 2^−^^△△Ct^ method. The primers were synthesized by Sangon Biotech Co., Ltd. (Shanghai, China), and the sequences of primers were shown in Table 1.

### 2.12. 16S-rDNA Sequencing of Cecal Content

We extracted the genomic DNA of the cecum or fecal contents of mice by the E.Z.N.A.^®^ soil DNA Kit (Omega Bio-Tek, Norcross, GA, USA) kit to ensure that the purity and integrity of the DNA met the experimental requirements.

This study selected the universal primers 338F (5′-ACTCCTACGGGAGGCAGCAG-3′) and 806R (5′-GGACTACHVGGGTWTCTAAT-3′) of 16S-rDNA to amplify the V3-V4 hypervariable region. The obtained PCR products were detected by 2% agarose gel electrophoresis, and the PCR products were recovered by cutting gel with the AxyPrep DNA Gel Recovery kit (Axygen, Union City, CA, USA). After that, the PCR products were treated with the QuantiFluor™-ST (Promega, Madison, WI, USA) and used for detection and quantification. The Miseq library was established and sequenced by the Illumina Miseq PE300 platform (Illumina, SD, USA) according to the standard scheme of Shanghai Majorbio Bio-pharm Technology Co., Ltd. (Shanghai, China). The observed taxon (OTUs) was based on UPARSE software, according to 97% similarity clustering. On this basis, the R software package was used to analyze alpha-diversity and beta-diversity. In addition, the microbial composition at phylum and genus levels was analyzed. Wilcoxon rank-sum test was used to compare two groups of microorganisms with significant differences at the genus level.

### 2.13. Data Analysis

All experimental data were expressed as mean ± SD, and the differences between each group were evaluated by one-way ANOVA and Tukey test. We used SPSS v20.0 software for statistical analysis and GraphPad prism8.0 for drawing. The value of *p* < 0.05 was considered a significant difference.

## 3. Results

### 3.1. Structural Characterization of BPP

**Figure 1:**
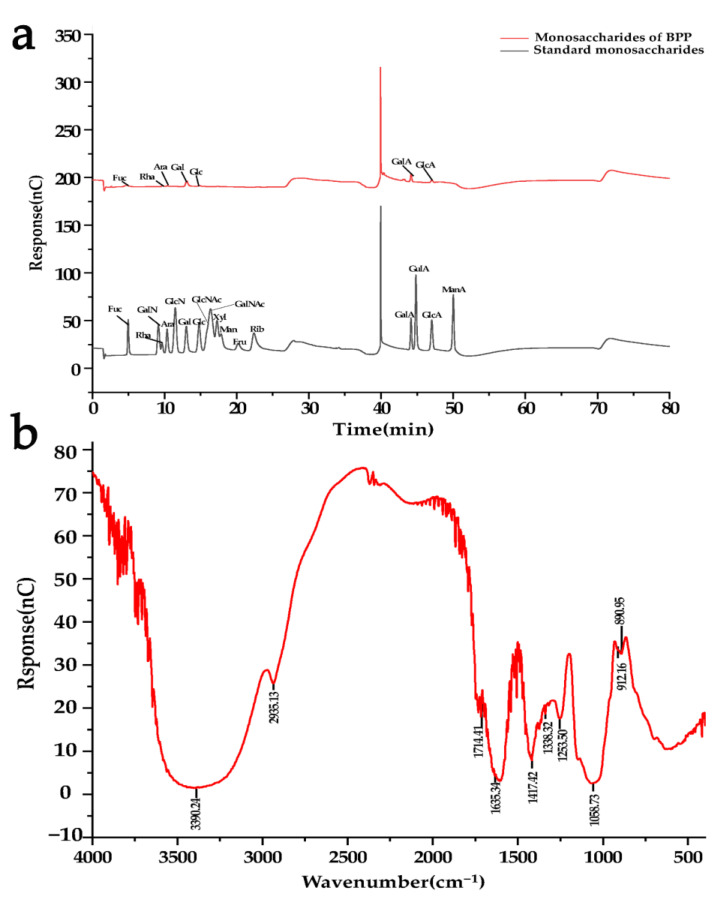
Structural characterization of BPP. (**a**) Molecular weight; (**b**) FT-IR spectrum analysis.

The standard curve established with glucose as the reference substance is y = 0.4963x + 0.1092 (R^2^ = 0.9966). There was no absorption peak at 260 nm or 280 nm, indicating no nucleic acid and protein in the BPP (Figure S1). Further study found that the BPP mainly consisted of galactose, galacturonic acid, fucose, rhamnose, glucuronic acid, glucose, and arabinose at a molar ratio of 28.2:27.9:15.0:12.4:9.3:3.9:3.3 (Figure 1a). The absorption band at 3600–3200 cm^−1^ is the stretching vibration absorption peak of -OH, and the absorption peak in this region is the characteristic peak of sugars. There was an absorption peak at 2935 cm^−1^, which may be attributed to C-H stretching vibration. There was an absorption peaked at 1714 cm^−1^, attributed to C=O stretching vibration. An absorption peak that appeared at 1635 cm^−1^ might be due to crystalline water. An absorption peak arose at 1417 cm^−1^, which might belong to C-O stretching vibration. There was an absorption peak at 1338 cm^−1^, indicating C=O symmetric stretching vibration. Absorption peaks emerged at 1253 cm^−1^ and 1058 cm^−1^, which were related to O-H variable angle vibration. There was an absorption peak at 912 cm^−1^, which may be attributed to the asymmetric ring stretching vibration of the pyran ring. An absorption peak was at 890 cm^−1^, which might confirm the structure of the pyran ring β-C-H variable angle vibration of the terminal group differential isomerism (Figure 1b). These results showed that BPP had characteristic adsorption of typical polysaccharides.

### 3.2. BPP Improved APAP Induced Liver Dysfunction

**Figure 2:**
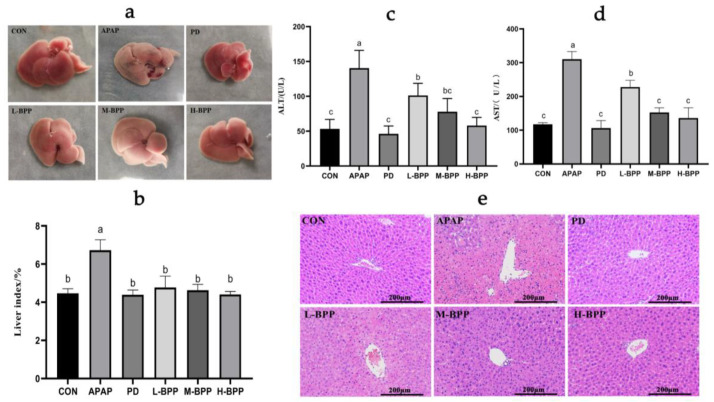
BPP ameliorated APAP-induced liver injury in mice. (**a**) Liver morphology. (**b**) Liver index. (**c**) Serum ALT levels. (**d**) Serum AST levels. (**e**) Liver pathological section. Data are expressed as mean ± SD. Different letters indicate statistically significant differences (*p* < 0.05) between the groups.

The liver of normal mice was reddish-brown and soft. In the APAP group, the liver color was uneven and we found necrosis and spots on the surface while the liver index increased. In the L-BPP, M-BPP, and H-BPP groups, the liver appearance was improved in varying degrees, and the liver index decreased significantly (Figure 2a,b and Table S1). Compared with the control group, the activities of ALT and AST in serum of the APAP group increased significantly, indicating that the liver of mice was damaged. In addition, the serum ALT and AST levels after BPP treatment were significantly lower than those in the APAP group (Figure 2c,d). In addition, APAP causes serious structural changes such as loose arrangement of hepatocytes, blood extravasation, vacuolar degeneration and obvious necrosis (Figure 2e). The liver structural damage was alleviated after treatment with BPP and bifendate, and the efficacy of the M-BPP and H-BPP groups seemed equivalent to that of the PD group.

### 3.3. BPP Inhibited APAP-Induced Liver Oxidative Stress

**Figure 3:**
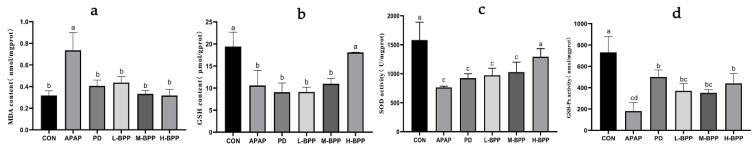
Hepatic MDA, GSH, SOD and GSH-Px content or activities. (**a**) MDA. (**b**) GSH. (**c**) SOD. (**d**) GSH-Px. Data are expressed as mean ± SD. Different letters indicate statistically significant differences (*p* < 0.05) between the groups.

APAP caused oxidative stress in the liver compared with the CON group, such that MDA levels increased significantly, and GSH, GSH-Px, and SOD levels decreased significantly. After BPP treatment, the MDA level in the liver of mice was restored, and the GSH, GSH-Px, and SOD levels were also increased. BPP alleviated the oxidative stress of mice liver induced by APAP (Figure 3). Surprisingly, M-BPP and H-BPP groups could achieve or even exceed the effect of the PD group.

### 3.4. BPP Enhanced Expression of Nrf2 and Its Target Genes

**Figure 4:**
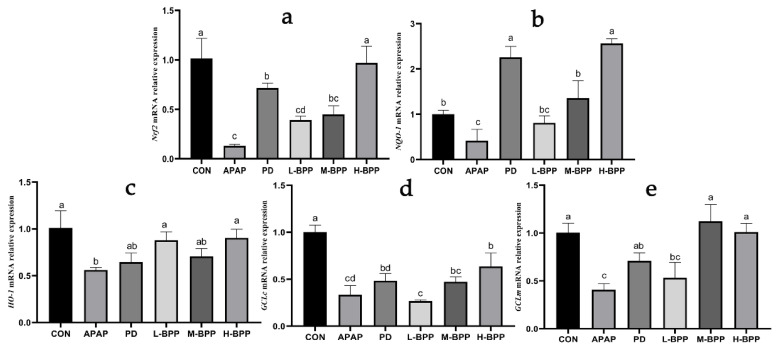
Quantitative analysis of Nrf2, NQO-1, HO-1, GCLc and GCLm. (**a**) Nrf2. (**b**) NQO-1. (**c**) HO-1. (**d**) GCLc. (**e**) GCLm. Data are expressed as mean ± SD. Different letters indicate statistically significant differences (*p* < 0.05) between the groups.

Nrf2 plays a crucial role in resisting oxidative stress and is considered a potential therapeutic target for acute liver injury. We found that APAP could significantly reduce the mRNA levels of Nrf2 and its target genes, including NQO-1, HO-1, GCLc, and GCLm. High doses of BPP (400 mg/kg) increased the mRNA levels of Nrf2 and its target genes (including NQO-1, HO-1, GCLm, and GCLc) (Figure 4). In particular, the role of the H-BPP group is even more helpful than the PD group.

### 3.5. Effects of BPP on Major Metabolic Enzymes in Liver of Mice with Liver Injury

**Figure 5:**
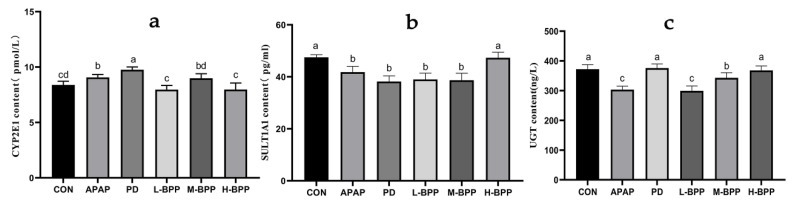
Changes of main metabolic enzymes in liver of mice with liver injury. (**a**) CYP2E1. (**b**) SULT1A1. (**c**) UGT. Data are expressed as mean ± SD. Different letters indicate statistically significant differences (*p* < 0.05) between the groups.

APAP could increase CYP2E1 levels and aggravate liver toxicity. Early administration of BPP doses of 100 and 400 mg kg^−1^ decreased the content of CYP2E1 (Figure 5a). At the same time, BPP could increase the levels of SULT1A1 and UGT in a dose-dependent manner (Figure 5b,c). The above results suggested that BPP may reduce the accumulation of NAPQI by accelerating the detoxification ability of the liver to APAP. Moreover, the effect of the H-BPP group was better than that of the PD group.

### 3.6. BPP Alleviated Liver Cell Apoptosis Induced by APAP

**Figure 6:**
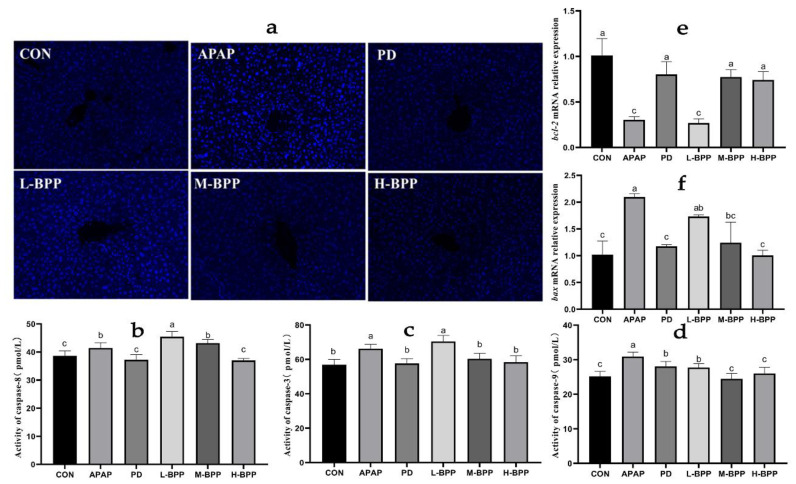
Effect of BPP on liver cell apoptosis induced by APAP. (**a**) Hoechst 33258. (**b**) caspase-8. (**c**) caspase-3. (**d**) caspase-9. Quantitative analysis of bcl-2 (**e**) and bax (**f**). Data are expressed as mean ± SD. Different letters indicate statistically significant differences (*p* < 0.05) between the groups.

Hoechst 33258 staining results showed that the APAP group showed dense bright blue, indicating that a large of cells had died, which disappeared after 200 and 400 mg/kg BPP treatment (Figure 6a). Moreover, APAP increased the activity of caspase-3/8/9, and BPP pretreatment could change the enzyme activity related to apoptosis (Figure 6b–d). In addition, M-BPP and H-BPP regulated the abnormal mRNA levels of bax and bcl-2 induced by APAP (Figure 6e,f). Surprisingly, the effect of the H-BPP group on reducing apoptosis was similar to that of the PD group.

### 3.7. BPP Reduced Inflammatory Factors in the Liver

**Figure 7:**
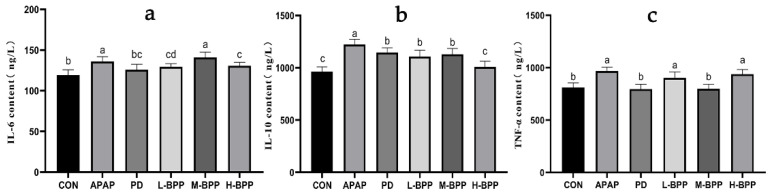
Effect of BPP on inflammatory factors in APAP induced liver injury mice. (**a**) IL-6. (**b**) IL-10. (**c**) TNF-α. Data are expressed as mean ± SD. Different letters indicate statistically significant differences (*p* < 0.05) between the groups.

Excessive inflammatory factors can aggravate liver injury. As shown in Figure 7, the levels of IL-6 and IL-10 increased significantly after APAP administration. It was worth noting that compared with the CON group, APAP also increased the content of IL-10 (*p* < 0.05). Compared with the APAP group, the levels of IL-6, IL-10, and TNF-α in the liver of mice treated with BPP in advance were decreased. In addition, we also found that the IL-10 levels of L-BPP and M-BPP were significantly higher than those of the CON group, but the level of the H-BPP was close to that of CON group.

### 3.8. BPP Improved the Structure of Intestinal Microflora in Injured Mice

Sobs and Ace indexes reflected the richness of the community. The richness of the H-BPP group was significantly higher than that of the APAP and CON groups (*p* < 0.05), suggesting that BPP could increase the number of intestinal microorganisms. Shannon and Simpson indexes reflected the diversity of the microbial community. The Simpson index of the APAP group decreased significantly, and the Shannon index increased significantly (*p* < 0.05), indicating that the diversity of the community increased after APAP treatment, which might be caused by the proliferation of some harmful bacteria (Table S2).

**Figure 8:**
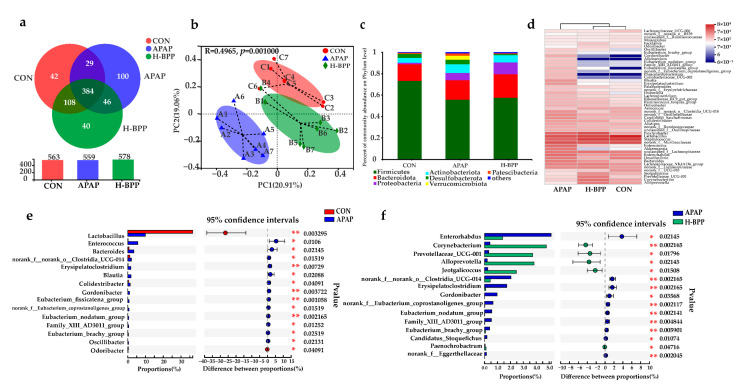
Characteristics of intestinal microbiota in mice after APAP and BPP treatment. (**a**) Species Venn diagram analysis OTU number. (**b**) Principal Component Analysis (PCoA) of gut microbiota at OUTs level. (**c**) Relative abundance of microbiota at the phylum level. (**d**) Relative abundance of microbiota at the genus level. (**e**) Difference of dominant species between CON group and APAP group at genus level. (**f**) Difference of dominant species between APAP group and H-BPP group at genus level. * *p* < 0.05, ** *p* < 0.01.

Figure 8a indicated that the CON group, APAP groups, and H-BPP groups shared 384 OTUs, showing the existence of a vital core microbiota. The CON and H-BPP groups shared 108 OTUs, but the CON and APAP groups only shared 29 OTUs. Principal coordinate analysis (PCoA) showed that the community composition of the H-BPP group was closer to that of the CON group than that of the APAP group (Figure 8b). Figure 8c showed the community composition at the phylum level among different groups. Further analysis found that the distribution of bacteria at the phylum level showed that the relative abundance of Deferribacterota increased significantly and the relative abundance of Firmicutes decreased significantly after APAP treatment (*p* < 0.05). The relative abundance of Desulfobacterota and Deferibacterota in the H-BPP group was lower than that of mice in the APAP group, but the abundance of Bdellovibrionota increased significantly (*p* < 0.05).

Figure 8d showed the community composition at the genus level among different groups. Through heatmap cluster analysis, it was obvious that the community composition of the H-BPP group was more similar to that of the CON group. Figure 8e,f showed the significant differences among the three groups at the genus level. *Lactobacillus* and *Odoribacter* decreased significantly after APAP treatment (*p* < 0.05). The abundance of *Enterococcus*, *Bacteroides*, *norank_f_norank_o_ Clostridia_UCG-014*, *Erysipelatoclostridium*, *Blautia*, *Colidextribacter*, *Gordonibacter*, *Eubacterium_fissicatena_group*, *norank_f_Eubacterium_coprostanoligenes_group*, *Eubacterium _nodatum_group*, *Family_XIII_AD3011_group*, *Eubacterium_brachy_ group* and *Oscillibacter* increased significantly (*p* < 0.01 or *p* < 0.05). As shown in Figure 8f, the abundance of *Enterorhabdus*, *norank_f_norank_o_Clostridia_UCG-014*, *Erysipelatoclostridium*, *Gordonibacter*, *norank_f_Eubacterium_coprostanoligenes_group*, *Eubacterium_nodatum_group*, *Family_XIII_AD3011_group*, *Eubacterium_brachy_group*, *Candidatus_Stoquefichus* and *norank_f_Eggerthellaceae* of H-BPP group was significantly reduced compared with the APAP group. Meanwhile, the abundance of *Corynebacterium*, *Prevotellaceae_UCG-001*, *Alloprevotella*, *Jeotgalicoccus*, and *Paenochrobactrum* in the caecum of the H-BPP group increased significantly (*p* < 0.01 or *p* < 0.05). It is noteworthy that at the genus level, the flora of 13 genera increased significantly after APAP treatment, and 7 genera recovered to varying degrees after BPP treatment. In short, the composition of the intestinal flora of BPP pretreated mice was more reasonable and alleviated the intestinal flora disorder caused by APAP.

### 3.9. FMT Alleviated the Symptoms of Liver Injury in APAP-Induced Mice

**Figure 9:**
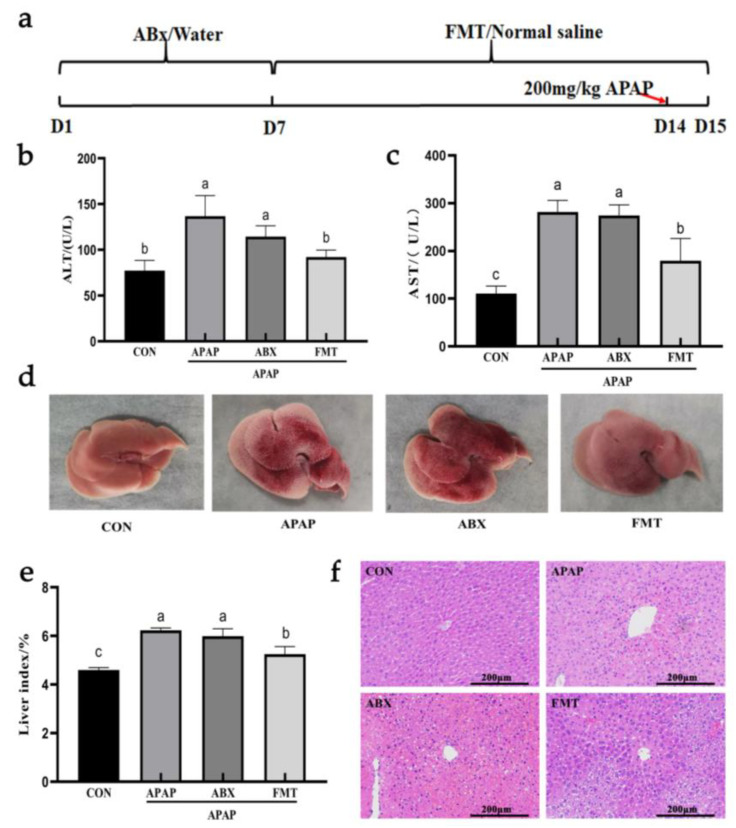
FMT ameliorated APAP-induced liver injury in mice. (**a**) FMT procedure. (**b**) Liver morphology. (**c**) Serum ALT levels. (**d**) Serum AST levels. (**e**) Liver index. (**f**) Liver pathological section (H&E). Data are expressed as mean ± SD. Different letters indicate statistically significant differences (*p* < 0.05) between the groups.

We reconstructed the new intestinal flora structure according to the procedure described in Figure 9a. Mice in the APAP group and ABX group showed similar symptoms of liver injury after APAP treatment. After fecal microbiota transplantation, serum ALT and AST levels in mice were significantly lower than those in the ABX group, especially ALT levels close to the CON group (Figure 9b,c). After FMT treatment, the morphology of mice liver was restored visually, and the liver index was partially restored (Figure 9d,e and Table S3). These results of pathological tissue sections showed that most of the liver structures of mice were normal after FMT treatment. However, there were still some pathological changes around the central vein, which was consistent with the results of liver morphology (Figure 9f). In conclusion, reconstituting the intestinal flora of mice could resist the liver injury caused by APAP.

### 3.10. Changes of Intestinal Microbiota in Mice after ABX and FMT

**Figure 10:**
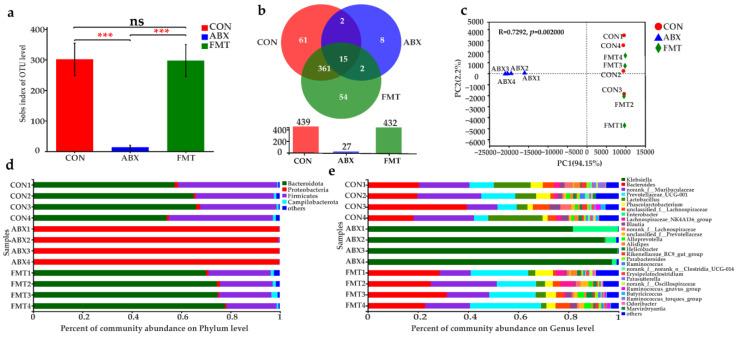
Characterization of the gut microbiota in mice after Abx treatment and FMT. (**a**) Index group difference test based on Student’s *t* test. (**b**) Species Venn diagram analysis OTU number. (**c**) Principal Component Analysis (PCA) of gut microbiota. (**d**) Relative abundance of microbiota at the phylum level. (**e**) Relative abundance of microbiota at the genus level. The “ns” indicated there was no significant difference between the groups (*p* > 0.05). *** *p* < 0.001.

The results of 16S rDNA sequencing of mouse feces aere shown in Figure 10. The α diversity analysis showed that the number of microorganisms in mouse feces in the ABX group decreased sharply (*p* < 0.0001), and the number of the FMT and CON groups was similar (Figure 10a). Figure 10b shows that the ABX group had only 27 OUTs, while the CON and FMT groups had 439 and 432 OUTs. The CON group and FMT groups shared 376 common OUTs. Figure 10c shows that the positions of the ABX group, CON group, and FMT group are significantly different in the PCA diagram, indicating that the species composition of the ABX group had changed considerably, while the CON group and FMT group were similar. The microbial community composition at phylum and genus levels also fully showed that the community composition was reduced after antibiotic treatment (Figure 10d,e). The above results showed that the clearance of intestinal flora in mice was successful, and a new intestinal flora structure was reconstructed after fecal microbiota transplantation.

At the phylum level, Firmicutes, Bacteroidota, and Actinobacteriota were the dominant flora in the CON and FMT groups, which accounted for more than 90% of all the flora. However, in the ABX group, Bacteroidota and Actinobacteriota accounted for only 0.25% and 99% of Proteobacteria. At the genus level, the dominant floras of the ABX group were *Klebsiella* (93.19%) and *Enterobacter* (6.09%). The dominant bacteria in the CON group were *Bacteroides* (24.48%) and *norank_f_Muribaculaceae* (20.46%), *Lactobacillus* (12.23%), *Prevotellaceae, UCG-001* (9.17%). The dominant bacteria in the FMT group were *Bacteroides* (27.03%) and *norank_f_Muribaculaceae* (18.36%), *Prevotellaceae_UCG-001* (21.39%). Comparing the different species composition at the genus level, *Prevotellaceae_UCG-001*, *norank _f_mitochondrial*, *unclassified_o_Bacteroidales* in the FMT group were significantly higher than those in CON group (*p* < 0.05 or *p* < 0.01), which might be the main reason why FMT could resist APAP-induced liver injury.

### 3.11. Evaluation of the Liver Protective Effect of BPP on Pseudo Sterile Mice

**Figure 11:**
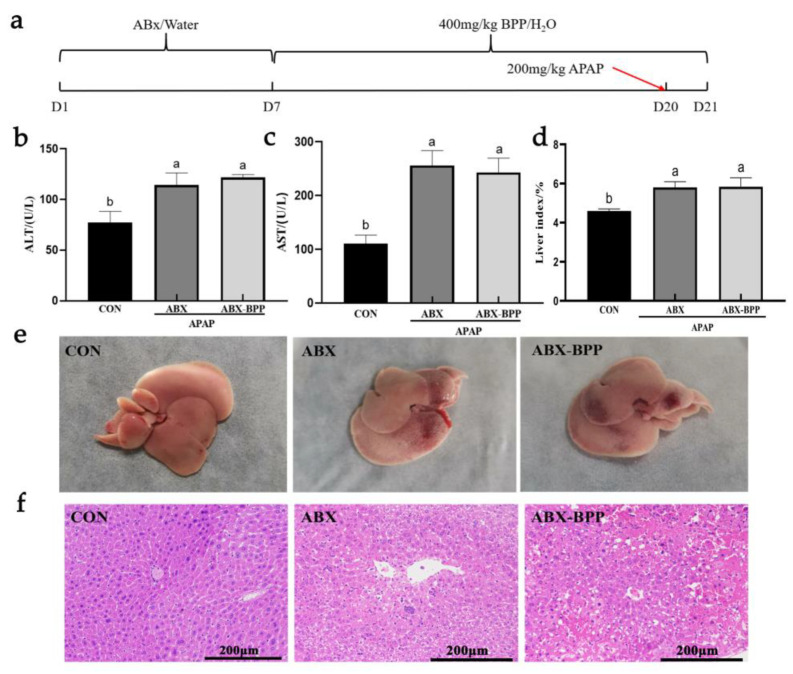
Effect of BPP on mice with gut microbiota depletion (**a**) Experimental procedure. (**b**) Serum ALT levels. (**c**) Serum AST levels. (**d**) Liver index. (**e**) Liver morphology. (**f**) Liver pathological section (H&E). Data are expressed as mean ± SD. Different letters indicate statistically significant differences (*p* < 0.05) between the groups.

According to the procedure in Figure 11a, intestinal flora was first cleared, and then 400 mg/kg BPP was administered for 14 days. The serum ALT and AST in ABX and ABX-BPP groups were significantly higher than those in the CON group (Figure 11b,c). The liver index showed similar changes (Figure 11d and Table S4). The liver in the ABX and ABX-BPP groups showed visible liver bleeding, uneven color, and obvious surface spots (Figure 11e).

Pathological examination of the liver showed that the structure of the hepatic cord in the ABX group was fuzzy, and the liver cells had obvious necrosis and hemorrhage. In addition to the above changes, mice in the ABX-BPP group also had some vacuolization of liver cells (Figure 11f). In conclusion, BPP could not protect the liver from APAP damage after depletion of gut microbiota.

## 4. Discussion

At present, APAP-induced liver injury has become the most popular and clinically relevant model for testing phytotherapeutic agents and other liver-protective interventions [29]. Both serum ALT and AST are typical biochemical indicators that can be used to assess liver function [30]. In this study, serum ALT and AST levels increased significantly after APAP administration, indicating that liver injury had occurred in mice. The pathological changes in the liver also confirmed it. However, BPP pretreatment was shown to effectively decrease the elevated levels of ALT and AST, indicative of the restoration of hepatic function. APAP caused severe damage to the hepatic architecture, resulting in serious centrilobular hepatic necrosis and inflammatory infiltration, which BPP significantly ameliorated. These results suggest that BPP has the potential to prevent APAP-induced acute liver injury.

As is known to all, excessive APAP damages the liver because a large amount ofhepatotoxic NAPQI is produced under the action of CYP2E1 [31]. Therefore, inhibiting CYP2E1 activity or its expression can reduce liver injury by reducing the formation of NAPQI. We found that BPP could inhibit the hyperactivity of CYP2E1 and increase the activity of UGT and SULT. These changes will accelerate the transformation of APAP to non-toxic compounds, which may be one of the mechanisms that reduced liver injury.

A large amount of evidence showed that mitochondrial oxidative stress is the primary cellular event after APAP poisoning, and the levels of GSH, GSH Px, SOD, and MDA could reflect the oxidation of the body [32]. In our study, BPP treatment upregulated levels of antioxidant proteins, including SOD, GSH-Px, and GSH, thus inhibiting oxidative stress in APAP-induced liver injury. Nrf2 knockout mice have increased sensitivity to APAP-induced liver injury, suggesting that Nrf2 is a potential target for treating APAP-induced liver injury [33]. Previous reports have shown that tovophyllin A and Guavinoside B prevented APAP-induced hepatotoxicity by activating the Nrf2 signaling pathway [34,35]. Similarly, our study found that BPP treatment upregulated Nrf2 expression and its downstream genes NQO-1, HO-1, GCLm, and GCLc. This activation of the Nrf2 pathway might explain the alleviating effect of BPP on hepatic oxidative stress induced by APAP.

NAPQI is the primary mechanism of liver injury, including the formation of protein adducts through reaction with sulfhydryl groups, leading to mitochondrial dysfunction and cell death. Cell contents released after cell death will stimulate the body to produce an inflammatory response. In the early stage of liver injury, this process can effectively remove dead cells and fragments. However, excessive inflammation will inevitably lead to more severe damage and even liver cell death. This study found that IL-6, TNF-α, and IL-10 in the APAP group were significantly increased. IL-10 is usually considered to be an anti-inflammatory cytokine which can inhibit the production and response of pro-inflammatory cytokines in innate and adaptive immunity. However, APAP-induced liver injury could also lead to an abnormal increase in IL-10 content because the production of IL-10 was related to the time and degree of liver injury [36].

At the same time, the inflammatory cytokines that APAP induces will cause further cell apoptosis. Three enzymes (caspase-3, caspase-8, and caspase-9) that participate in the apoptosis process were detected in the APAP-treated mice [37]. It has already been proved that an APAP overdose increased the protein expressions of bax, decreased bcl-2 protein expression, and initiated the activation of caspase-3, caspase-8, and caspase-9 [38]. Therefore, increasing the ratio of bcl-2/bax could reduce the APAP-induced apoptosis [39,40]. After treatment with BPP, the mRNA level of bcl-2 in mice liver was increased, and the expression level of bax was decreased. At the same time, the contents of caspase-3, caspase-8, and caspase-9 proteins in the liver of mice treated with BPP also decreased, indicating that apoptosis was reduced, which was consistent with the results of H&E staining and Hoechst 33258 staining. Therefore, we speculated that the anti-apoptotic biological activity of BPP might be one of the reasons for resisting APAP-induced liver injury.

APAP leads to the abnormality of intestinal flora, and the structure of intestinal flora also affects the liver. By supplementing exogenous probiotics or probiotics to change the system of intestinal flora, metabolites of intestinal microbes (such as short-chain fatty acids and bile acids) act on the liver through the “gut-liver axis”, which plays a key role in drug-induced hepatotoxicity [10]. Oral *Lactobacillus rhamnosus* GG has been shown to protect against APAP-induced liver injury by activating Nrf2 via *Lactobacillus*-derived 5-methoxyindoleacetic acid [41]. Recent reports have shown that oral administration of *Akkermansia muciniphila* improved oxidative stress and inflammation to protect against APAP-induced liver injury, which was closely related to the remodeling of intestinal microflora and enhanced short-chain fatty acids (SCFAs) [42]. In this study, the hepatoprotective effect of BPP disappeared after depleting the intestinal flora. Furthermore, the symptoms of APAP-induced liver injury were alleviated after reconstructing the intestinal flora structure by FMT. Although the serum AST and liver structure of the FMT group were still different from those of the normal mice, we proved that intestinal flora played an essential role in protecting of APAP-induced liver injury by BPP.

The results of this study confirmed that BPP improved the diversity of the microbial community and regulated the relative abundance of dominant microbiota at the phylum and genus level. We found the relative abundance of Firmicutes decreased significantly after APAP treatment, which is consistent with previous reports [5]. In addition, previous studies showed that the content of isovaleric acid in serum increased after APAP treatment, and Deferribacta was positively correlated with the production of isovaleric acid [43,44]. Desulfobacterota and Deferribactota have been shown to be associated with intestinal inflammation, which may partly explain the anti-inflammatory effect of BPP [45]. There was evidence that Bdellovibrionota was positively correlated with the content of GSH, but the specific mechanism is not clear [46]. At the genus level, *Lactobacillus* decreased significantly after APAP treatment, which was consistent with the existing reports [6]. *Lactobacillus* and *Odoribacter* were beneficial to the production of SCFA, and their abundance was reduced after APAP supplementation, thus causing and promoting inflammatory response [47]. Therefore, APAP might play a pro-inflammatory role by regulating specific intestinal microbiota and its metabolites.

At the genus level, the flora of 13 genera increased significantly after APAP treatment, and the 7 genera recovered to varying degrees after BPP treatment, including *norank_f_norank_o_Clostridia_UCG-014*, *Erysipelatoclostridium*, *Gordonibacter*, *norank_f_Eubacterium_coprostanoligenes_group*, *Eubacterium_nodatum_group*, *Family_XIII_AD3011_group*, and *Eubacterium_brachy_group*. These bacteria are closely related to liver injury or metabolism [47,48,49,50,51]. Furthermore, BPP increased the richness of *Corynebacterium*, *Prevotellaceae_UCG-001*, *Alloprevotella,* and *Jeotgalicoccus*, which was similar to the report that walnut green peel polysaccharide and Spirulina platensis polysaccharides have a hepatoprotective effect [11,52]. *Prevotellaceae_UCG-001* is involved in the decomposition of polysaccharides such as degrading inulin [53]. It also could produce SCFAs, including acetate and butyrate, thus exerting anti-inflammatory effects on immune cells and inhibiting the growth of potential invasive pathogens [54]. In addition, *Prevotellaceae* is usually enriched due to the treatment of polysaccharides, which is associated with the transforming growth factor β3 that regulates the intestinal barrier function [55]. After fecal microbiota transplantation, the content of *Prevotellaceae_UCG-001* was significantly increased compared with the CON group. We speculated that the bacterium might play an important role in protecting mice from APAP liver injury.

## 5. Conclusions

In summary, the current research results provided strong evidence for the hepatoprotective effect of BPP. BPP could reduce serum AST and ALT levels, protect the liver from damage by APAP, and enhance the antioxidant capacity of the liver. In addition, BPP could also reduce the level of liver inflammation and hepatocyte apoptosis. Surprisingly, BPP has better liver protection effects than positive drugs in some aspects. These results implied that the mechanism of action of BPP on the liver might be through the activation of the Nrf2 pathway, inhibition of APAP metabolism, and regulation of apoptosis. More importantly, we demonstrated that the hepatoprotective effect of BPP was closely related to the remodeled intestinal flora. BPP might be explored as an effective functional component to resist liver injury caused by APAP and associated metabolic diseases.

## Figures and Tables

**Table 1:** Primer sequence.

Gene Name	Primer Sequence (5′-3′)	Accession Number
β-Actin	F: GTGCTATGTTGCTCTAGACTTCG R: ATGCCACAGGATTCCATACC	NM_007393.5
Nrf2	F: TAGATGACCATGAGTCGCTTGC R: GCCAAACTTGCTCCATGTCC	NM_010902.4
HO-1	F: AAGCCGAGAATGCTGAGTTCA R: GCCGTGTAGATATGGTACAAGGA	NM_010442.2
NQO-1	F: AGGATGGGAGGTACTCGAATC R: AGGCGTCCTTCCTTATATGCTA	NM_008706.5
GCLc	F: GGGGTGACGAGGTGGAGTA R: GTTGGGGTTTGTCCTCTCCC	NM_010295.2
GCLm	F: CGGGAACCTGCTCAACTG R: CCAAAACATCTGGAAACTCCC	NM_008129.4
bcl-2	F: GCTACCGTCGTGACTTCGC R: CCCCACCGAACTCAAAGAAGG	NM_009741.5
bax	F: AGACAGGGGCCTTTTTGCTAC R: AATTCGCCGGAGACACTCG	XM_011250780.4

## Data Availability

Not applicable.

## Supplementary Materials

The following supporting information can be downloaded at: https://www.mdpi.com/article/10.3390/nu14132636/s1, Figure S1: UV scanning spectrum of BPP; Table S1. Effect of BPP on liver index in mice; Table S2. Alpha diversity index table; Table S3. Effect of FMT on liver index in mice; Table S4. Effect of BPP on liver index in pseudo sterile mice.
