# Supplementary material for: Broussonetia papyrifera Polysaccharide Alleviated Acetaminophen-Induced Liver Injury by Regulating the Intestinal Flora
Source: Nutrients. 2022 Jun 25;14(13):2636. doi: 10.3390/nu14132636 (PMC9268590; doi:10.3390/nu14132636)
Supplement: Supplementary file 1 [file nutrients-1763147-supplementary.pdf › nutrients-1763147-supplementary.pdf]

Supplementary Materials

Figure S1:

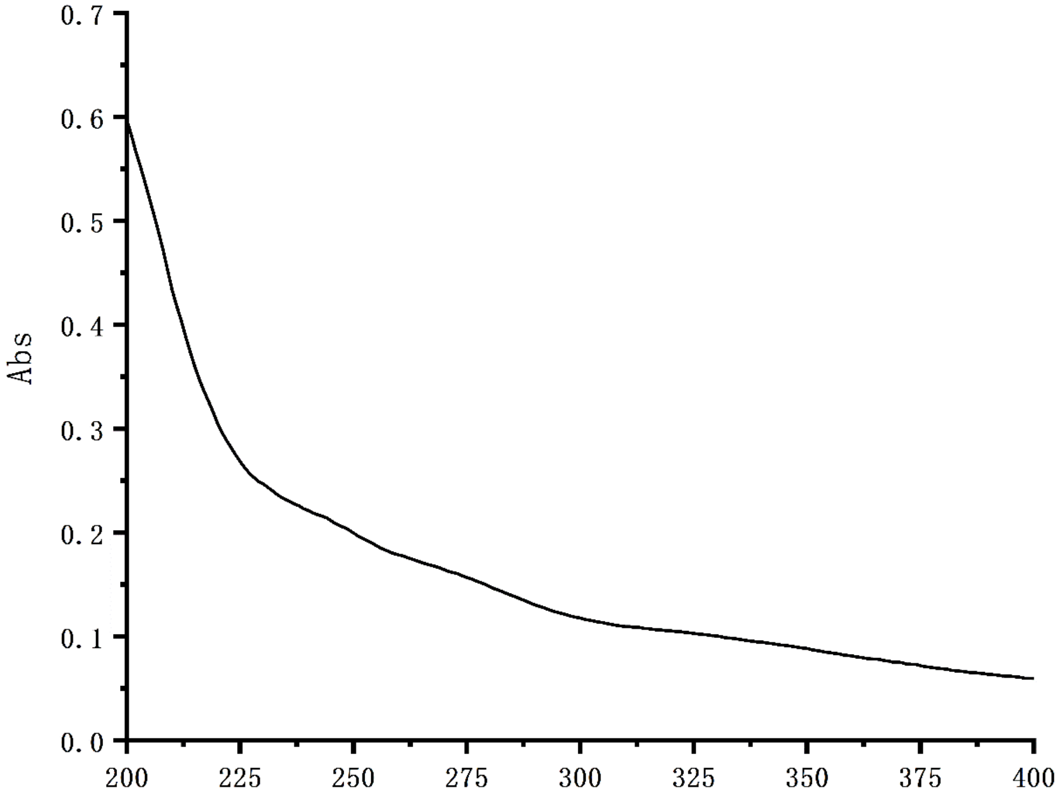

Figure S1 UV scanning spectrum of BPP

Table S1

Table S1. Effect of BPP on liver index in mice

Groups	Body weight (g)	Liver weight (g)	Liver index (%)
CON	27.75±1.74[b]	1.24±0.08[b]	4.46±0.25[b]
APAP	23.6±1.33[c]	1.59±0.16[a]	6.73±0.55[a]
PD	27.89±1.82[b]	1.22±0.12[b]	4.39±0.25[b]
L-BPP	30.29±2.46[ab]	1.44±0.12[a]	4.77±0.6[b]
M-BPP	33.16±3.34[a]	1.54±0.22[a]	4.63±0.3[b]
H-BPP	31.38±1.32[a]	1.38±0.1[b]	4.41±0.16[b]

Different data markers in the same column indicate significant differences ($p < 0.05$)

Table S2

Table S2 Alpha diversity index table

Groups	CON	APAP	H-BPP
sobs	318.14±43.017^{b}	303.57±25.98^{b}	331.43±34.534^{a}
ace	374.71±41.166^{a}	345.34±35.104^{a}	385.6±34.344^{a}
shannon	3.221±0.626^{b}	3.827±0.206^{a}	3.493±0.543^{b}
simpson	0.124±0.075^{a}	0.055±0.01^{b}	0.088±0.051^{a}

Table S3

Table S3. Effect of FMT on liver index in mice

Groups	Body weight (g)	Liver weight (g)	Liver index (%)
CON	29.73±2.17^{b}	1.37±0.09^{c}	4.6±0.1^{c}
APAP	27.75±0.91^{a}	1.73±0.05^{a}	6.23±0.1^{a}
ABX	27.28±0.76^{a}	1.63±0.08^{b}	5.99±0.31^{a}
FMT	28.13±1.51^{ab}	1.48±0.12^{c}	5.25±0.32^{b}

Different data markers in the same column indicate significant differences ($p < 0.05$)

Table S4

Table S4. Effect of BPP on liver index in pseudo sterile mice

Groups	Body weight (g)	Liver weight (g)	Liver index (%)
CON	29.73±2.17^{a}	1.37±0.09^{a}	4.6±0.1^{b}
ABX	28.18±0.76^{a}	1.63±0.08^{b}	5.8±0.3^{a}
ABX-BPP	27.54±1.39^{b}	1.6±0.12^{b}	5.83±0.46^{a}

Different data markers in the same column indicate significant differences ($p < 0.05$)
